# COVID-19 Sequelae and the Host Proinflammatory Response: An Analysis From the OnCovid Registry

**DOI:** 10.1093/jnci/djac057

**Published:** 2022-04-13

**Authors:** Alessio Cortellini, Alessandra Gennari, Fanny Pommeret, Grisma Patel, Thomas Newsom-Davis, Alexia Bertuzzi, Margarita Viladot, Juan Aguilar-Company, Oriol Mirallas, Eudald Felip, Alvin J X Lee, Alessia Dalla Pria, Rachel Sharkey, Joan Brunet, MCarmen Carmona-García, John Chester, Uma Mukherjee, Lorenza Scotti, Saoirse Dolly, Ailsa Sita-Lumsden, Daniela Ferrante, Mieke Van Hemelrijck, Charlotte Moss, Beth Russell, Elia Seguí, Federica Biello, Marco Krengli, Javier Marco-Hernández, Gianluca Gaidano, Andrea Patriarca, Riccardo Bruna, Elisa Roldán, Laura Fox, Anna Pous, Franck Griscelli, Ramon Salazar, Clara Martinez-Vila, Anna Sureda, Angela Loizidou, Clara Maluquer, Annabelle Stoclin, Maria Iglesias, Paolo Pedrazzoli, Gianpiero Rizzo, Armando Santoro, Lorenza Rimassa, Sabrina Rossi, Nadia Harbeck, Ana Sanchez de Torre, Bruno Vincenzi, Michela Libertini, Salvatore Provenzano, Daniele Generali, Salvatore Grisanti, Rossana Berardi, Marco Tucci, Francesca Mazzoni, Matteo Lambertini, Marco Tagliamento, Alessandro Parisi, Federica Zoratto, Paola Queirolo, Raffaele Giusti, Annalisa Guida, Alberto Zambelli, Carlo Tondini, Antonio Maconi, Marta Betti, Emeline Colomba, Nikolaos Diamantis, Alasdair Sinclair, Mark Bower, Isabel Ruiz-Camps, David J Pinato, OnCovid study group, Georgina Hanbury, Chris Chung, Meera Patel, Gino Dettorre, Christopher C T Sng, Tamara Yu, Marianne Shawe-Taylor, Hamish D C Bain, Lee Cooper, Lucy Rogers, Katherine Belessiotis, Cian Murphy, Samira Bawany, Saira Khalique, Ramis Andaleeb, Eleanor Apthorp, Roxana Reyes, David Garcia-Illescas, Nadia Saoudi, Ariadna Roqué Lloveras, Ricard Mesia, Andrea Plaja, Marc Cucurull, Federica Grosso, Vittorio Fusco, Alice Baggi, Maristella Saponara, Luca Cantini

**Affiliations:** Department of Surgery and Cancer, Imperial College London, Hammersmith Hospital, London, UK; Division of Oncology, Department of Translational Medicine, University of Piemonte Orientale, Novara, Italy; Department of Cancer Medicine, Institut Gustave Roussy, University of Paris Saclay, Villejuif, France; Cancer Division, University College London Hospitals, London, UK; Department of Oncology and National Centre for HIV Malignancy, Chelsea and Westminster Hospital, London, UK; Medical Oncology and Hematology Unit, Humanitas Cancer Center, IRCCS Humanitas Research Hospital, Rozzano, Milan, Italy; Department of Medical Oncology, Hospital Clinic, Barcelona, Spain; Medical Oncology, Vall d'Hebron University Hospital and Institute of Oncology (VHIO), Barcelona, Spain; Infectious Diseases, Vall d'Hebron University Hospital, Barcelona, Spain; Medical Oncology, Vall d'Hebron University Hospital and Institute of Oncology (VHIO), Barcelona, Spain; Medical Oncology Department, B-ARGO Group, IGTP, Catalan Institute of Oncology, Badalona, Spain; Cancer Division, University College London Hospitals, London, UK; Department of Oncology and National Centre for HIV Malignancy, Chelsea and Westminster Hospital, London, UK; Department of Oncology and National Centre for HIV Malignancy, Chelsea and Westminster Hospital, London, UK; Department of Medical Oncology, Catalan Institute of Oncology, University Hospital Josep Trueta, Girona, Spain; Department of Medical Oncology, Catalan Institute of Oncology, University Hospital Josep Trueta, Girona, Spain; Medical Oncology, School of Medicine, Cardiff University, Cardiff, UK; Medical Oncology, Velindre Cancer Centre, Cardiff, UK; Medical Oncology, Barts Health NHS Trust, London, UK; Department of Translational Medicine, Unit of Medical Statistics, University of Piemonte Orientale, Novara, Italy; Medical Oncology, Guy’s and St Thomas’ NHS Foundation Trust (GSTT), London, UK; Medical Oncology, Guy’s and St Thomas’ NHS Foundation Trust (GSTT), London, UK; Department of Translational Medicine, Unit of Medical Statistics, University of Piemonte Orientale, Novara, Italy; Translational Oncology and Urology Research (TOUR), School of Cancer and Pharmaceutical Sciences, King’s College London, London, UK; Translational Oncology and Urology Research (TOUR), School of Cancer and Pharmaceutical Sciences, King’s College London, London, UK; Translational Oncology and Urology Research (TOUR), School of Cancer and Pharmaceutical Sciences, King’s College London, London, UK; Department of Medical Oncology, Hospital Clinic, Barcelona, Spain; Division of Oncology, Department of Translational Medicine, University of Piemonte Orientale, Novara, Italy; Division of Radiotherapy, Department of Translational Medicine, University of Piemonte Orientale and Azienda Ospedaliera Maggiore Della Carita, Novara, Italy; Department of Medical Oncology, Hospital Clinic, Barcelona, Spain; Division of Haematology, Department of Translational Medicine, University of Piemonte Orientale and Maggiore della Carità Hospital, Novara, Italy; Division of Haematology, Department of Translational Medicine, University of Piemonte Orientale and Maggiore della Carità Hospital, Novara, Italy; Division of Haematology, Department of Translational Medicine, University of Piemonte Orientale and Maggiore della Carità Hospital, Novara, Italy; Infectious Diseases, Vall d'Hebron University Hospital, Barcelona, Spain; Hematology Department, Vall d'Hebron Institute of Oncology (VHIO), Vall d’Hebron Hospital Universitari, Barcelona, Spain; Medical Oncology Department, B-ARGO Group, IGTP, Catalan Institute of Oncology, Badalona, Spain; Department of Biology and Pathology, Gustave Roussy Cancer Campus, Villejuif, France; Faculté des Sciences Pharmaceutiques et Biologiques, Université de Paris, Sorbonne Paris Cité, Paris, France; Department of Medical Oncology, ICO L’Hospitalet, Oncobell Program (IDIBELL), CIBERONC, Hospitalet de Llobregat, Barcelona, Spain; Fundació Althaia Manresa, Manresa, Spain; Haematology Department, ICO Hospitalet, Hospitalet de Llobregat, IDIBELL, Universitat de Barcelona, Barcelona, Spain; Department of Infectious Diseases, Internal Medicine, Institut Jules Bordet, Université Libre de Bruxelles, Brussels, Belgium; Haematology Department, ICO Hospitalet, Hospitalet de Llobregat, IDIBELL, Universitat de Barcelona, Barcelona, Spain; Department of Cancer Medicine, Institut Gustave Roussy, University of Paris Saclay, Villejuif, France; Hospital Son Llatzer, Palma de Mallorca, Spain; Medical Oncology Unit, Fondazione IRCCS Policlinico San Matteo, Pavia, Italy; Department of Internal Medicine and Medical Therapy, University of Pavia, Pavia, Italy; Medical Oncology Unit, Fondazione IRCCS Policlinico San Matteo, Pavia, Italy; Medical Oncology and Hematology Unit, Humanitas Cancer Center, IRCCS Humanitas Research Hospital, Rozzano, Milan, Italy; Department of Biomedical Sciences, Humanitas University, Pieve Emanuele, Milan, Italy; Medical Oncology and Hematology Unit, Humanitas Cancer Center, IRCCS Humanitas Research Hospital, Rozzano, Milan, Italy; Department of Biomedical Sciences, Humanitas University, Pieve Emanuele, Milan, Italy; Medical Oncology and Hematology Unit, Humanitas Cancer Center, IRCCS Humanitas Research Hospital, Rozzano, Milan, Italy; Department of Gynecology and Obstetrics, Breast Center and Gynecological Cancer Center and CCC Munich, University Hospital Munich, Munich, Germany; Hospital Universitario XII de Octubre, Madrid, Spain; Policlinico Universitario Campus Bio-Medico, Rome, Italy; Medical Oncology Unit, Fondazione Poliambulanza Istituto Ospedaliero, Brescia, Italy; Medical Oncology 2, Fondazione IRCCS Istituto Nazionale dei Tumori, Milan, Italy; Multidisciplinary Breast Pathology and Translational Research Unit, ASST Cremona, Cremona, Italy; Department of Medical, Surgical and Health Sciences, University of Trieste, Trieste, Italy; Medical Oncology Unit, Spedali Civili, Brescia, Italy; Medical Oncology, AOU Ospedali Riuniti, Polytechnic University of the Marche Region, Ancona, Italy; Section of Medical Oncology, Department of Biomedical Sciences and Clinical Oncology (DIMO), University of Bari ‘Aldo Moro’, Bari, Italy; IRCCS, Istituto Tumori Giovanni Paolo II, Bari, Italy; Medical Oncology, Careggi University Hospital, Florence, Italy; Medical Oncology Department, IRCCS Ospedale Policlinico San Martino, Genova, Italy; Department of Internal Medicine and Medical Specialties (DiMI), School of Medicine, University of Genova, Genova, Italy; Medical Oncology Department, IRCCS Ospedale Policlinico San Martino, Genova, Italy; Department of Internal Medicine and Medical Specialties (DiMI), School of Medicine, University of Genova, Genova, Italy; Department of Life, Health and Environmental Sciences, University of L’Aquila, L’Aquila, Italy; Medical Oncology, St Maria Goretti Hospital, Latina, Italy; Melanoma and Sarcoma Medical Treatment Unit, IEO—Istituto Europeo di Oncologia, Milan, Italy; Medical Oncology, St. Andrea Hospital, Rome, Italy; Department of Oncology, Azienda Ospedaliera Santa Maria, Terni, Italy; Oncology Unit, ASST Papa Giovanni XXIII, Bergamo, Italy; Oncology Unit, ASST Papa Giovanni XXIII, Bergamo, Italy; Infrastruttura Ricerca Formazione Innovazione, Azienda Ospedaliera SS Antonio e Biagio e Cesare Arrigo, Alessandria, Italy; Infrastruttura Ricerca Formazione Innovazione, Azienda Ospedaliera SS Antonio e Biagio e Cesare Arrigo, Alessandria, Italy; Department of Cancer Medicine, Institut Gustave Roussy, University of Paris Saclay, Villejuif, France; Department of Translational Medicine, Unit of Medical Statistics, University of Piemonte Orientale, Novara, Italy; Cancer Division, University College London Hospitals, London, UK; Department of Oncology and National Centre for HIV Malignancy, Chelsea and Westminster Hospital, London, UK; Infectious Diseases, Vall d'Hebron University Hospital, Barcelona, Spain; Department of Surgery and Cancer, Imperial College London, Hammersmith Hospital, London, UK; Division of Oncology, Department of Translational Medicine, University of Piemonte Orientale, Novara, Italy

## Abstract

**Background:**

Fifteen percent of patients with cancer experience symptomatic sequelae, which impair post–COVID-19 outcomes. In this study, we investigated whether a proinflammatory status is associated with the development of COVID-19 sequelae.

**Methods:**

OnCovid recruited 2795 consecutive patients who were diagnosed with Severe Acute Respiratory Syndrome Coronavirus 2 infection between February 27, 2020, and February 14, 2021. This analysis focused on COVID-19 survivors who underwent a clinical reassessment after the exclusion of patients with hematological malignancies. We evaluated the association of inflammatory markers collected at COVID-19 diagnosis with sequelae, considering the impact of previous systemic anticancer therapy. All statistical tests were 2-sided.

**Results:**

Of 1339 eligible patients, 203 experienced at least 1 sequela (15.2%). Median baseline C-reactive protein (CRP; 77.5 mg/L vs 22.2 mg/L, *P* < .001), lactate dehydrogenase (310 UI/L vs 274 UI/L, *P* = .03), and the neutrophil to lymphocyte ratio (NLR; 6.0 vs 4.3, *P* = .001) were statistically significantly higher among patients who experienced sequelae, whereas no association was reported for the platelet to lymphocyte ratio and the OnCovid Inflammatory Score, which includes albumin and lymphocytes. The widest area under the ROC curve (AUC) was reported for baseline CRP (AUC = 0.66, 95% confidence interval [CI]: 0.63 to 0.69), followed by the NLR (AUC = 0.58, 95% CI: 0.55 to 0.61) and lactate dehydrogenase (AUC = 0.57, 95% CI: 0.52 to 0.61). Using a fixed categorical multivariable analysis, high CRP (odds ratio [OR] = 2.56, 95% CI: 1.67 to 3.91) and NLR (OR = 1.45, 95% CI: 1.01 to 2.10) were confirmed to be statistically significantly associated with an increased risk of sequelae. Exposure to chemotherapy was associated with a decreased risk of sequelae (OR = 0.57, 95% CI: 0.36 to 0.91), whereas no associations with immune checkpoint inhibitors, endocrine therapy, and other types of systemic anticancer therapy were found.

**Conclusions:**

Although the association between inflammatory status, recent chemotherapy and sequelae warrants further investigation, our findings suggest that a deranged proinflammatory reaction at COVID-19 diagnosis may predict for sequelae development.

Increasing evidence highlights that an important proportion of COVID-19 survivors are at risk of protracted symptomatic consequences after the acute Severe Acute Respiratory Syndrome Coronavirus 2 (SARS-CoV-2) infection (1). This condition, termed “long COVID-19,” is a clinically recognized syndrome of likely immune-inflammatory pathogenesis (2) with broad health-care and societal implications (3). A wide variety of COVID-19 sequelae have been described so far, including respiratory symptoms and functional impairment, persisting fatigue, and neuro-cognitive changes, with a duration of symptoms that can extend beyond 6 months postinfection (4).

In the general population, between 13% and 60% of COVID-19 survivors are at risk of developing post–COVID-19 symptoms (4-7). Considering the intrinsic vulnerability of patients with cancer, these figures are concerning for their potential impact on the resumption of active anticancer therapy and surveillance after COVID-19 recovery.

The OnCovid study, the largest European COVID-19 and cancer registry (8–13), has highlighted that at least 15% of COVID-19 survivors with cancer experience medium- or long-term symptoms, including most frequently respiratory sequelae (49.6%) and fatigue (41.0%) (14). Most importantly, we showed that the emergence of COVID-19 sequelae was associated with a statistically significant worsening of patient survival and with a lower likelihood of resuming systemic anticancer therapy (SACT) (14).

Against this background, the lack of biomarkers that can predict for the future emergence of COVID-19 sequelae in patients who survive the acute phase is a point of the utmost interest. The discovery of reproducible clinical and biologic predictors is an area of high unmet need, because it would allow clinicians to identify patient subgroups who should be prioritized for enhanced follow-up, preventative strategies, and therapeutic interventions.

Inflammation is a recognized driver of severe COVID-19 also in patients with cancer (15); we previously showed that the systemic proinflammatory response identifies patients experiencing adverse outcomes in the OnCovid study population (13). However, the relationship between the systemic proinflammatory response and the onset of COVID-19 sequelae is unknown.

The purpose of this study is to verify whether noninvasive biomarkers of the systemic inflammatory response measured at SARS-CoV-2 infection diagnosis are associated with the emergence of sequelae in patients who survive COVID-19.

## Methods

### Study Population, Setting, and Data Collection

OnCovid (NCT04393974) is an active European registry study enrolling consecutive patients fulfilling the following inclusion criteria: 1) aged 18 years and older; 2) diagnosis of SARS-CoV-2 infection confirmed by reverse transcription-polymerase chain reaction (RT-PCR) of a nasopharyngeal swab (16); and 3) history of solid or hematologic malignancy at any time during patients’ past medical history, either active or in remission at the time of COVID-19 diagnosis.

For the purpose of this analysis, we focused on patients who survived COVID-19 and underwent a formal clinical post–COVID-19 assessment at participating institutions (14).

Methodology of data collection for the OnCovid registry was described elsewhere (8,10,11), and sequelae definition, prevalence, assessment, and distribution were already detailed (14). In brief, COVID-19 sequelae were defined as any residual symptoms and/or measurable organ dysfunction attributable to COVID-19, following the criteria published by the World Health Organization (Supplementary Methods, available online) (17).

The study population was accrued from 35 institutions across 6 countries (UK, Italy, Spain, France, Belgium, and Germany) and diagnosed with COVID-19 between February 27, 2020, and February 14, 2021. The data lock for the present analysis was March 1, 2021.

### Study Endpoints and Definitions

The primary objective of this study was to evaluate a panel of proinflammatory biomarkers of consolidated prognostic role in patients with cancer (18) measured at the time of COVID-19 diagnosis and evaluate them for their association with the development of COVID-19–related sequelae during routine oncological follow-up after COVID-19 recovery. Patients with hematological malignancies were excluded, in view of the potential confounding effect of the underlying oncological disease in the computation of bone marrow–derived inflammatory parameters.

Inflammatory indices were evaluated at COVID-19 diagnosis according to the clinical practice of participating centers and included C-reactive protein (CRP; mg/L), lactate dehydrogenase (LDH; UI/L), the neutrophil to lymphocyte ratio (NLR), the platelet to lymphocyte ratio (PLR), and the OnCovid Inflammatory Score (OIS), which combines lymphopenia and hypoalbuminemia only (albumin concentration [g/L] + 5 × total lymphocyte count [10^9^/L] as a derivation of the prognostic nutritional index, already renamed in the context of COVID-19) (13).

Before any clinicopathologic correlation, we first reported the distribution of each biomarker and then evaluated their individual predictive ability for the association with COVID-19 sequelae as continuous variables through receiver operating characteristic (ROC) analyses. Acknowledging that the effect of inflammatory indices in the post–COVID-19 setting had not been investigated before, we also computed optimal cutoffs to test them as categorical variables in fixed multivariable models for COVID-19 sequelae overall, respiratory sequalae, and post–COVID-19 fatigue. Considering missing data for laboratory values and their scattered distribution, each inflammatory biomarker was evaluated independently. An exploratory ROC curves comparison was also performed. We also evaluated the impact on post–COVID-19 survival of inflammatory biomarkers assessed at the time of first oncologic reassessment, which included the laboratory testing with remote clinical consultations or face-to-face visits.

Accounting for the unbalanced distribution of patient- and disease-related features across the subgroups, we used fixed multivariable regression models, adjusting all estimates for clinical characteristics already known to influence outcomes in patients with COVID-19 and cancer and as already performed in previously published analyses from the OnCovid study (8,10–12). Key factors used as adjusting covariates are detailed in the Supplementary Methods (available online). Considering our previous results, the time from cancer diagnosis to post–COVID-19 reassessment was not included as a covariate (9). Moreover, although OnCovid was not powered to report on individual country-level estimates, country was also used as adjusting factor (United Kingdom; Spain; Italy; and France, Belgium, or Germany) (10).

In addition, we evaluated the relationship between COVID-19 sequelae and exposure to different types of SACT at COVID-19 diagnosis. Patients who were not on SACT were elected as the reference group. Exposure to SACT was defined as the receipt of any SACT regimen within 4 weeks before SARS-CoV-2 infection diagnosis and was categorized as follows: chemotherapy (including chemotherapy alone and as combinations with other agents and/or immune checkpoint inhibitors [ICIs]); ICI-based regimens (without chemotherapy); endocrine therapy; tyrosine kinase inhibitors (TKIs) and monoclonal antibodies (MABs); and poly adenosine diphosphate-ribose polymerase inhibitors (PARPi) and cyclin dependent kinase 4/6 inhibitors (CDK4/6i).

In consideration of the complex interrelationships between SACT and the underlying tumor in influencing clinical outcomes in cancer patients diagnosed with COVID-19, the interaction terms between SACT regimens and primary tumor, tumor stage, and tumour status were also tested in independent models, and separate additional analyses among patients with advanced and nonadvanced disease were presented. Lastly, we tested the distribution of median baseline inflammatory markers or indices statistically significantly associated with COVID-19 sequelae according to different SACT regimens. Detailed study methodology is summarized in Supplementary Methods (available online).

OnCovid was granted central approval by the United Kingdom Health Research Authority (20/HRA/1608) and by the corresponding research ethics committees at each participating institution. A full waiver of consent because of the retrospective nature of the study was granted by the UK Health Research Authority in accordance with UK law because of the anonymized nature of the patient data and retrospective design of the study.

### Statistical Analysis

Baseline characteristics were summarized as categorical variables and reported using descriptive statistics. Associations between categorical variables were tested using the Pearson χ^2^ test. Inflammatory markers were reported as median with interquartile ranges (IQRs). The Kruskal–Wallis test was used to compare the median values of continuous data. A ROC curve analysis with the computation of the area under the curve (AUC) was performed for each inflammatory marker with respect of COVID-19 sequelae, then the optimal cutoffs were determined using the Youden’s J statistic. Fixed multivariable logistic regression models were used to assess the impact of analyzed factors on the risk of COVID-19 sequelae and presented as odds ratios (ORs) with 95% confidence intervals (CIs). Through the fixed models, each inflammatory index was evaluated separately but adjusting for all the same preselected covariates. Post–COVID-19 survival was defined as the length of time from the date of the first post–COVID-19 assessment to the date of a patient’s death (for any cause) or last follow-up and was estimated with the Kaplan–Meier method, with comparisons computed with the log-rank test. The median post–COVID-19 follow-up was estimated with the reverse Kaplan-Meier method. Multivariable Cox proportional hazards models were used to assess the impact on the risk of death after COVID-19 recovery and were presented as hazard ratios (HRs) with 95% confidence intervals. To verify hazards distribution proportionality, the interaction with time of each inflammatory marker at the post–COVID-19 reassessment was tested. *P* less than .05 was considered statistically significant, and all statistical tests were 2-sided. Analyses were performed using the MedCalc Statistical Software version 20 (MedCalc Software Ltd, Ostend, Belgium; https://www.medcalc.org; 2021) and the IBM SPSS Statistics software, Version: 28.0.1.0 (142). Figures were created in Prism V.8 (GraphPad, San Diego, CA, USA).

## Results

**Figure 1.**
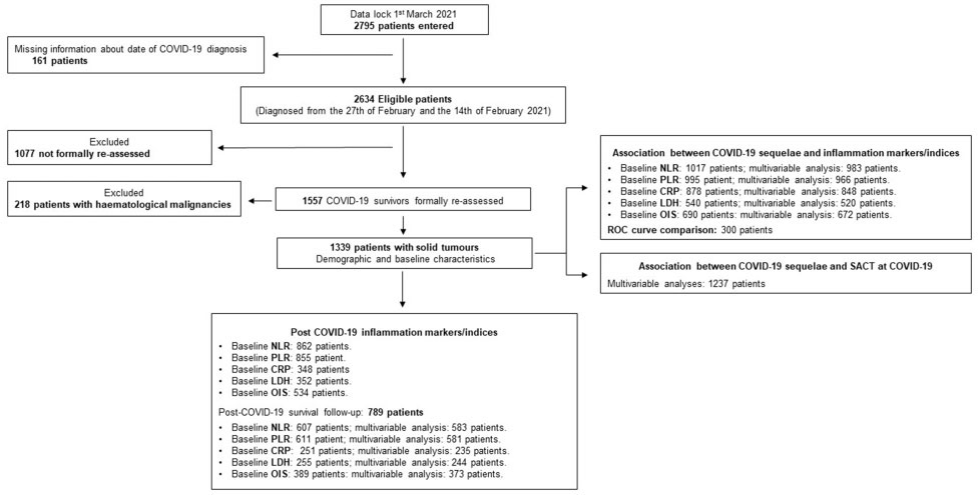
Study flow diagram. CRP = C-reactive protein; LDH = lactate dehydrogenase; NLR = neutrophil to lymphocyte ratio; OIS = OnCovid Inflammatory Score; PLR = platelet to lymphocyte ratio; ROC = receiver operating characteristics; SACT = systemic anticancer therapy.

As previously reported (14), 2795 consecutive patients were entered into the registry by the data lock. Of 2634 eligible patients, 1557 (59.1%) COVID-19 survivors were reassessed at participating institutions, and after the exclusion of 218 patients with hematological malignancies (14.1%), the final study population for this analysis consisted of 1339 patients evaluated after a median of 44 days (IQR = 27-63 days) post–COVID-19. Figure 1 provides a detailed study flow diagram with patients’ disposition across the planned analyses according to data availability, and patient distribution across participating centres is reported in Supplementary Table 1 (available online).

Overall, at least 1 sequela was reported for 203 patients (15.2%), including respiratory symptoms (97/203, 47.8%), residual fatigue (89/234, 43.8%), weight loss (13/203, 6.4%), neuro-cognitive symptoms (14/203, 6.9%), and others (47/203, 23.1%). The median times from COVID-19 to the post–COVID-19 reassessment according to the experience of sequelae were 42 days (IQR = 27-63.7 days) and 44 days (IQR = 27-64 days) (*P* = .78). Baseline patient, tumor, and COVID-19 characteristics of the included population overall and according to the experience of COVID-19 sequelae are reported in Supplementary Table 2 (available online).

Median baseline values of inflammatory markers (at COVID-19 diagnosis) among the overall population and according to COVID-19 sequelae are reported in Table 1. Median baseline CRP (77.5 mg/L vs 22.2 mg/L, *P* < .001), LDH (310 UI/L vs 274 UI/L, *P* = .03), and NLR (6.0 vs 4.3, *P* = .001) were statistically significantly higher among patients who experienced sequelae, whereas no association were reported regarding median baseline PLR and OIS. ROC curve analyses are summarized in Supplementary Figure 1 (available online); the widest AUC was reported for baseline CRP both when analyzed independently (AUC = 0.66, 95% CI = 0.63 to 0.69) and compared with other baseline markers (AUC = 0.66, 95% CI = 0.61 to 0.71), followed by the NLR (AUC = 0.58, 95% CI = 0.55 to 0.61) and LDH (AUC = 0.57, 95% CI = 0.52 to 0.61). Optimal cutoffs for baseline inflammatory markers or indices and their relevant categorical distribution according to sequelae are reported in Table 1. When analyzed as dichotomous variables, all the parameters were statistically significantly associated with the emergence of COVID-19 sequelae on univariate analysis.

**Table 1.** Median baseline values of inflammatory markers or indices at COVID-19 diagnosis among the overall population and according to COVID-19 sequelae^a^

Inflammatory markers	Overall study population (N = 1339)	Without COVID-19 Sequelae (n = 1136)	With COVID-19 Sequelae (n = 203)	*P*
	No. (%)	No. (%)	No. (%)	
Baseline CRP				
No. of patients	878	722	156	
Median (IQR), mg/L	28.9 (7.2-100.0)	22.2 (6.0-86.0)	77.5 (19.9-155.6)	< .001^b^
<36.7 mg/L	470 (53.5)	422 (58.4)	48 (30.8)	<.001^c^
≥36.7 mg/L	408 (46.5)	300 (41.4)	108 (69.2)	
Baseline LDH				
No. of patients	540	430	110	
Median (IQR), UI/L	281 (209-404)	274 (103-396)	310 (225-465)	.03^b^
<463 UI/L	449 (83.1)	368 (85.6)	81 (73.6)	.001^c^
≥463 UI/L	91 (16.9)	62 (14.4)	29 (26.4)	
Baseline NLR				
No. of patients	1017	837	180	
Median (IQR)	4.6 (2.5-9.1)	4.3 (2.4-8.7)	6.0 (3.0-11.7)	.001^b^
<5.7	589 (57.9)	506 (60.5)	83 (46.1)	.001^c^
≥5.7	428 (42.1)	331 (39.5)	97 (53.9)	
Baseline PLR				
No. of patients	995	820	175	
Median (IQR)	242 (151-392)	240 (153-302)	258 (145-396)	.69^b^
<455	815 (81.9)	681 (83.0)	134 (76.6)	.04^c^
≥455	180 (18.1)	139 (17.0)	41 (23.4)	
Baseline OIS				
No. of patients	690	569	121	
Median (IQR)	36 (31-40)	36 (31-40)	35 (31.7-40)	.35^b^
≥42	104 (15.1)	93 (16.3)	11 (9.1)	.04^c^
<42	586 (84.9)	476 (83.7)	110 (90.9)	

Categorical distribution is computed according to the individuated optimal cutoffs. CRP = C-reactive protein; IQR = interquartile range; LDH = lactate dehydrogenase; NLR = neutrophil to lymphocyte ratio; OIS = OnCovid Inflammatory Score; PLR = platelet to lymphocyte ratio.

Two-sided *P* values calculated with the Kruskal–Wallis test.

Two-sided *P* values calculated with the Pearson χ^2^ test.

**Figure 2.**
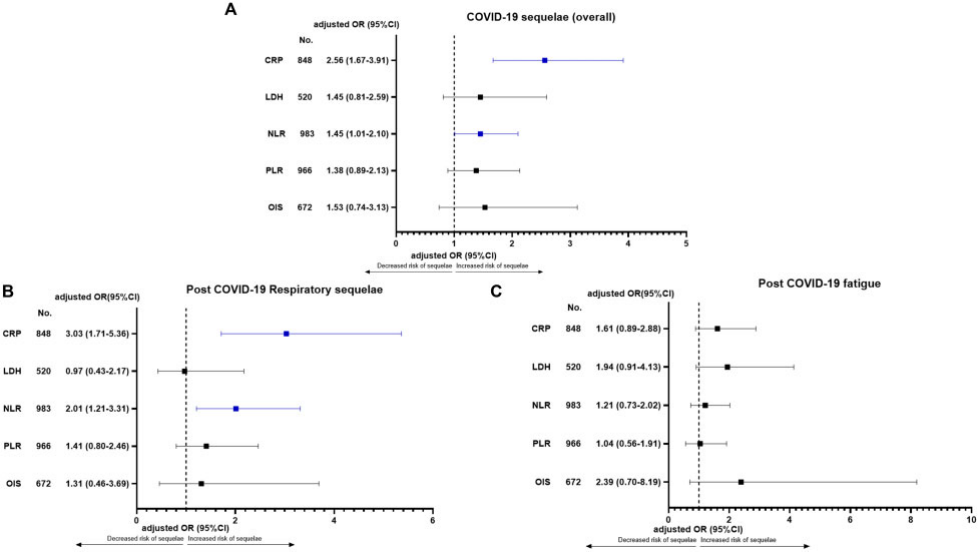
Fixed multivariable analysis according to categorized baseline inflammatory markers or indices for COVID-19 sequelae. **A**) COVID-19 sequelae overall, **B**) respiratory sequelae, and **C**) post–COVID-19 fatigue. Adjusting covariates for each analysis were sex (male vs female), age (≥65 vs <65 years), number of comorbidities (0-1 vs ≥2), primary tumor (clustered as breast, gastrointestinal, gynecological or genitourinary, thoracic, and others), receipt of systemic anticancer therapy within 4 weeks of COVID-19 diagnosis (yes vs no), tumor stage (defined as advanced vs nonadvanced), tumor status (presence of active vs nonactive disease), experience of at least 1 COVID-19 complication (yes vs no), receipt of any COVID-19–specific therapy (yes vs no), hospitalization (preexisting for whatever cause, including cancer vs due to COVID-19 vs not required), and country (United Kingdom; Spain; Italy; and France, Belgium, or Germany). CI = confidence interval; CRP = C-reactive protein; LDH = lactate dehydrogenase; NLR = neutrophil to lymphocyte ratio; OIS = OnCovid Inflammatory Score; OR = odds ratio; PLR = platelet to lymphocyte ratio.

Figure 2 reports the fixed multivariable analysis according to categorized inflammatory markers or indices at COVID-19 diagnosis for overall COVID-19 sequelae (Figure 2, A), respiratory sequelae (Figure 2, B), and post–COVID-19 fatigue (Figure 2, C). Increased CRP (OR = 2.56, 95% CI = 1.67 to 3.91) and NLR (OR = 1.45, 95% CI = 1.01 to 2.10) were confirmed to be statistically significantly associated with an increased risk of developing sequelae overall and respiratory sequelae (OR = 3.03, 95% CI = 1.71 to 5.36 and OR = 2.01, 95% CI = 1.21 to 3.31, respectively), whereas no association with post–COVID-19 fatigue was confirmed.

A further post–COVID-19 survival follow-up was available for 780 patients, with a median value of 123 days (95% CI = 103 to 147 days) and a median post COVID-19 survival, which was not reached (126 events) in the overall population.

Median values of post–COVID-19 inflammatory markers or indices were assessed at the same time as the clinical reassessment (median of 44 days from COVID-19), and their categorical distribution is summarized in Supplementary Table 3 (available online); Supplementary Figure 2 (available online) reports the Kaplan-Maier survival curves for post–COVID-19 survival according to CRP, LDH, NLR, PLR, and OIS. All post–COVID-19 markers were statistically significantly associated with post–COVID-19 survival on univariable analysis. Using fixed multivariable analysis reported in Supplementary Figure 3 (available online), increased post–COVID-19 CRP (HR = 3.18, 95% CI = 1.61 to 9.01), LDH (HR = 5.94, 95% CI = 2.19 to 16.07), NLR (HR = 3.29, 95% CI = 1.95 to 5.54), and a decreased OIS (HR = 3.27, 95% CI = 1.71 to 6.23) were statistically significantly associated with an increased risk of death, whereas no association was confirmed for post–COVID-19 PLR.

**Figure 3.**
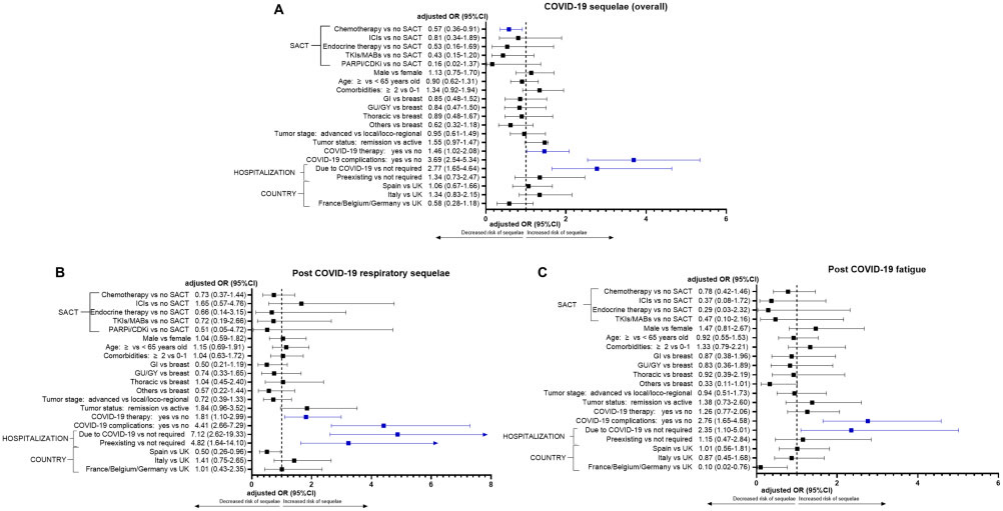
Fixed multivariable analysis including different type of systemic anticancer therapy (SACT) as covariate (1237 patients) for COVID-19 sequelae. **A**) COVID-19 sequelae overall, **B**) respiratory sequelae, and **C**) post–COVID-19 fatigue. CDKi = cyclin dependent kinase inhibitors; CI = confidence interval; GI = gastrointestinal; GU = genitourinary; GY = gynecological; ICIs = immune checkpoint inhibitors; MABs = monoclonal antibodies; OR = odds ratio; PARPi = poly adenosine diphosphate-ribose polymerase inhibitors; TKIs = tyrosine kinase inhibitors; UK = United Kingdom.

The proportions of patients receiving SACT within 4 weeks of COVID-19 diagnosis are reported in Supplementary Table 2 (available online). Overall, most patients were not receiving any SACT (870, 67.5%), followed by patients receiving chemotherapy (262, 20.3%), ICI-based regimens (53, 4.1%), endocrine therapy (32, 2.5%), TKIs or MABs (50, 3.9%), and PARPi or CDK4/6 inhibitors (22, 1.7%). Distribution of different types of SACT did not statistically significantly differ between patients who experienced COVID-19 sequelae and those who did not (*P* = .33). Figure 3 reports the fixed multivariable analysis for sequelae, including different types of SACT as covariate (1237 patients). The receipt of recent chemotherapy was associated with a decreased risk of sequelae overall (OR = 0.57, 95% CI = 0.36 to 0.91), whereas the receipt of COVID-19 therapy (OR = 1.46, 95% CI = 1.02 to 2.08), COVID-19 complications (OR = 3.69, 95% CI = 2.54 to 5.34), and hospitalization due to COVID-19 (OR = 2.77, 95% CI = 1.65 to 4.64) were confirmed to be associated with an increased risk (Figure 3, A). Regarding respiratory sequelae (Figure 3, B), and post–COVID-19 fatigue (Figure 3, B), none of the different SACT modalities was associated with risk of sequelae.

**Figure 4.**
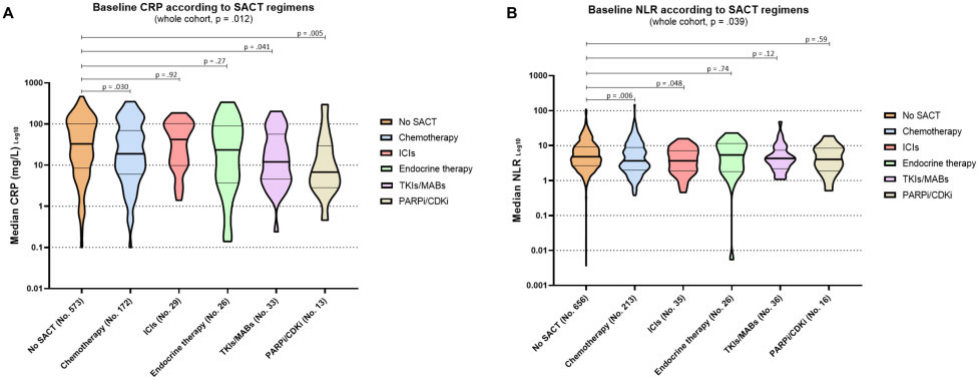
Median baseline inflammatory markers or indices according to different types of systemic anticancer therapy (SACT) regimens at COVID-19 diagnosis. **A**) C-reactive protein (CRP); no SACT: 33.0 mg/L (interquartile range [IQR] = 8.5-101.7 mg/L), chemotherapy = 18.8 mg/L (IQR = 6.1-68.8 mg/L), immune checkpoint inhibitors (ICIs) = 42.0 mg/L (IQR = 0.8-101.1 mg/L), endocrine therapy = 23.5 mg/L (IQR = 3.9-90 mg/L), tyrosine kinase inhibitors (TKIs) or monoclonal antibodies (MABs) = 12.0 mg/L (IQR = 4.6-53.5 mg/L), poly adenosine diphosphate-ribose polymerase inhibitors (PARPi) or cyclin dependent kinase inhibitors (CDKi) = 6.8 mg/L (IQR = 2.9-29.1 mg/L). **B**) Neutrophil to lymphocyte ratio (NLR); no SACT = 4.8 (IQR = 2.6-9.2), chemotherapy = 3.5 (IQR = 2.0-8.8), ICIs = 3.6 (IQR = 1.9-7.1), endocrine therapy = 5.4 (IQR = 1.8-11.3), TKIs or MABs = 4.2 (IQR = 2.2-6.9), PARPi or CDKi = 4.0 (IQR = 1.0-8.4). Median values are presented as Log_10_. Two-sided *P* values calculated with the Kruskal–Wallis test.

The median baseline CRP was statistically significantly different across different SACT categories (*P* = .012; Figure 4, A), with 18.8 mg/L for the chemotherapy group and 33.0 mg/L for the SACT group. Similarly, the median baseline NLR was also statistically significantly different across SACT categories (*P* = .04; Figure 4, B), with the highest value reported for the no SACT group (4.8) and the lowest value reported for the chemotherapy group (3.5).

Supplementary Table 4 (available online) reports the multivariable analyses, including the interaction terms between SACT regimens at COVID-19 and primary tumors (*P* = .06), tumor stage (*P* = .41), and tumor status (*P* = .82). The receipt of chemotherapy was confirmed to be associated with a decreased risk of sequelae among patients with nonadvanced disease on the multivariable analysis (OR = 0.37, 95% CI = 0.16 to 0.86) (Supplementary Figure 4, A, available online), whereas no association was reported among patients with advanced disease (OR = 0.77, 95% CI = 0.43 to 1.40) (Supplementary Figure 4, B, available online).

## Discussion

In the general population, persistent symptoms during the early postinfection phase are reported in over 36% of COVID-19 survivors (19), and up to 25% of patients may report sequelae beyond the 6-month landmark (6).

With countrywide immunization campaigns, enhanced hospital and testing capacity, and the availability of anti-SARS-CoV-2–specific therapies (12,20), an increasing proportion of patients with cancer now survive COVID-19, and investigating predictors of sequelae in this population is a clinical priority to facilitate proactive support strategies.

This study shows that routinely available inflammatory biomarkers measured at COVID-19 diagnosis, including CRP and NLR, are independently correlated with the emergence of sequelae, lending themselves as easily available tools to identify in advanced patients who is at higher risk of developing long COVID-19.

Although we recognize that certain COVID-19 sequelae—including, for instance, fatigue—may overlap with symptoms stemming from the underlying malignancy, previous studies have shown CRP to be reproducibly associated with respiratory impairment (21) and with COVID-19 severity in non–intensive care unit hospitalized patients (22), highlighting a mechanistic link following SARS-CoV-2 infection. CRP is in fact produced in response to interleukin-6 (IL-6) (23), one of the core mediators implicated in the cytokine release syndrome secondary to severe COVID-19 (24,25)

Regarding the NLR, neutrophilia and lymphopenia are already established markers of worse COVID-19 in the general population (26), and lymphopenia specifically seems to be another crucial step in the immunopathology of severe COVID-19. Excess systemic cytokine alters lymphopoiesis, producing lymphocytopenia in the peripheral blood alongside abnormal compensatory granulopoiesis (27). Immunocytometry analyses from severely ill patients with COVID-19 show an inversely proportional relationship between rising IL-6 serum levels and reduction of CD4 and CD8 peripheral T-cell counts, with evidence of an exhausted phenotype driven by the expression of PD-1 and Tim-3 (28).

In a previous study, we discovered that the presence of a sustained proinflammatory response at COVID-19 diagnosis, as evidenced by a combination of hypoalbuminemia and lymphocytopenia—termed the OIS—is the strongest determinant of mortality from COVID-19 (13), providing further evidence in support of the proinflammatory response as a mediator of adverse outcome in patients with COVID-19 and cancer.

In evaluating clinical factors associated with the development of COVID-19 sequelae, we concentrated on exposure to previous SACT in view of the differential influence of each individual therapeutic modality regarding the patient’s immune status (29). Interestingly, we report a statistically significantly lower proportion of COVID-19 sequelae in patients who had been exposed to chemotherapy within 4 weeks from COVID-19, a finding that is independent of major oncological features (primary tumor site, stage, and presence of active disease). Chemotherapy recipients were also those patients characterized by a statistically significantly lower median CRP and NLR, leading us to postulate whether the protective effect of chemotherapy on COVID-19 sequelae might be related to a therapy-dependent modulation of the proinflammatory response.

Although association cannot prove a causative link between chemotherapy and sequelae, emerging biologic plausibility between immune imbalances and post–COVID-19 syndrome is beginning to be appreciated. Increasing evidence supports that SARS-CoV-2–induced autoimmunity plays a role in the pathogenesis of the post–COVID-19 syndrome through eliciting autoantigen cross-reactivity, highlighted by the high prevalence of autoantibodies in SARS-CoV-2 convalescent serum (30). In addition, phenotypic alterations in B cells and T cells have been described in patients with COVID-19. Whereas B-cell changes, such as the reduction of transitional CD24^high^CD38^high^ cells, are largely restored in convalescent patients, T cells from recovered patients continue to show persistence of cytotoxic programming of CD8+ and elevated production of type 1 cytokines and IL-17 (31), which is known to be a driver of inflammatory reactions and autoimmunity (32).

Considering the rationale for the well-established use of immune suppressive agents such as IL-6 inhibitors (33) and corticosteroids (34) in specific phases of COVID-19, it is fascinating to speculate about a possible beneficial role of recent chemotherapy in reducing unopposed proinflammatory signalling through its broad immune-modulating effects (29). Our previous finding linking older age with reduced risk of COVID-19 sequelae in cancer patients poses immune-senescence and chemotherapy-induced immune suppression as 2 putative protective mechanisms against the proinflammatory signalling that drives SARS-CoV-2–related sequelae (14,35,36). However, considering the limited sample size of patient subgroups according to different SACT regimens, our findings allow only speculative reflections and should be taken with the utmost caution.

As highlighted in previously published reports from our registry (14), major study limitations for this update lie in the absence of predefined time points and techniques for sequelae assessment and in the inability of accounting for the role of SARS-CoV-2 vaccinations. In fact, only a minority of patients had received at least 1 dose by the data lock, and all of them after having already contracted the virus. We must also acknowledge the risk of selection bias and missing values for laboratory data leading to unavoidable attrition in evaluable patients with each biomarker available. In fact, baseline CRP and LDH were available for a considerably lower number of patients than the NLR, preventing proper comparisons of their predictive ability. For that reason, it is prudent to advocate for the equal use of both in routine practice.

Despite these limitations, our study provides clinically useful information regarding the diagnostic ability of routinely available inflammatory markers or indices to individuate patients at higher risk of developing COVID-19 sequelae, who should be prioritized for tailored follow-up procedures, proactive rehabilitation, and clinical trials with anti-inflammatory- and/or immune-modulating strategies (37-39). Interestingly, patients receiving chemotherapy within 4 weeks of COVID-19 diagnosis seem to be at decreased risk of developing sequelae, a finding that needs to be further investigated to fully elucidate its underlying mechanisms.

## Funding

OnCovid is sponsored by Imperial College London and received direct project funding and infrastructural support by the NIHR Imperial Biomedical Research Centre (BRC).

## Notes

**Role of the funder:** Neither sponsor nor the funders of the study had any role in study design, data collection, data analysis, data interpretation, or writing of the report.

**Disclosures:** DJP received lecture fees from ViiV Healthcare, Bayer Healthcare, BMS, Roche, EISAI, Falk Foundation, travel expenses from BMS and Bayer Healthcare; consulting fees for Mina Therapeutics, EISAI, Roche, DaVolterra and Astra Zeneca; research funding (to institution) from MSD and BMS. AP has declared personal honoraria from Pfizer, Roche, MSD Oncology, Eli Lilly, and Daiichi Sankyo; travel, accommodations, and expenses paid by Daiichi Sankyo; research funding from Roche and Novartis; and consulting/advisory role for NanoString Technologies, Amgen, Roche, Novartis, Pfizer and Bristol-Myers Squibb.

Matteo Lambertini acted as consultant for Roche, Novartis, Lilly, AstraZeneca, Exact Sciences, MSD, Pfizer, Seagen and received speaker honoraria from Roche, Novartis, Lilly, Pfizer, Takeda, Ipsen and Sandoz outside the submitted work. EF declared research founding to institution be Pfizer and travel expenses from Lilly, Novartis, Pfizer and Esai. TND has declared consulting/advisory role for Amgen, Bayer, AstraZeneca, BMS, Boehringer Ingelheim, Eli Lilly, MSD, Novartis, Otsuka, Pfizer, Roche, and Takeda; speakers fees from AstraZeneca, MSD, Roche, Takeda and travel, accommodations and expenses paid by AstraZenca, BMS, Boehringer Ingelheim, Lilly, MSD, Otsuka, Roche, and Takeda. JB has declared consulting/advisory role for MSD and Astra Zeneca. MT declares travel grants from Roche, Bristol-Myers Squibb, AstraZeneca, Takeda and Honoraria as medical writer from Novartis, Amgen outside the submitted work. AG has declared consulting/advisory role for Roche, MSD, Eli Lilly, Pierre Fabre, EISAI, and Daichii Sankyo; speakers bureau for Eisai, Novartis, Eli Lilly, Roche, Teva, Gentili, Pfizer, Astra Zeneca, Celgene, and Daichii Sankyo; research funds: EISAI, Eli Lilly, and Roche. CMV has received travel grants and other honoraria from BMS, MSD, Novartis and Roche. GG has declared consulting/advisory role for Janssen, Abbvie, Astra-Zeneca and BeiGene, and speaker fees from Janssen and Abbvie. LR received consulting fees from Servier, Amgen, ArQule, AstraZeneca, Basilea, Bayer, BMS, Celgene, Eisai, Exelixis, Genenta, Hengrui, Incyte, Ipsen, IQVIA, Lilly, MSD, Nerviano Medical Sciences, Roche, Sanofi, Zymeworks; lecture fees from AbbVie, Amgen, Bayer, Eisai, Gilead, Incyte, Ipsen, Lilly, Merck Serono, Roche, Sanofi; travel expenses from Ipsen; and institutional research funding from Agios, ARMO BioSciences, AstraZeneca, BeiGene, Eisai, Exelixis, Fibrogen, Incyte, Ipsen, Lilly, MSD, Nerviano Medical Sciences, Roche, Zymeworks.

AC received consulting fees from MSD, BMS, AstraZeneca, Roche; speakers’ fee from AstraZeneca, MSD, Novartis and Eisai. All remaining authors have declared no conflicts of interest.

**Author contributions:** Conceptualization: AC, DJP. Methodology: AC, DJP. Software: AC. Validation: AC, DJP. Formal analysis: AC, LS, DF. Investigation: AC, AG, DJP. Resources: AG, DJP. Data Curation: AC, AG, FP, EC, GP TND, AB, MV, JAC, OM, EF, AJCL, ADP, RS, JB, AC, JC, UM, LS, SD, ASL, DF, MVH, CM, BR, ES, FB, MK, JMH, GG, AP, RB, ER, LF, AP, FG, RS, CMV, AS, AL, CM, AS, MI, PP, GR, AS, LR, SR, NH, ASdT, BV, ML, SP, DG, SG, RB, MT, FM, ML, MT, AP, FZ, PQ, RG, AG, AZ, CT, AM, MB, EC, ND, AS, MB, IRC, DJP. Writing—Original Draft: AC, DJP. Writing—Review and Editing: AC, AG, FP, EC, GP TND, AB, MV, JAC, OM, EF, AJCL, ADP, RS, JB, AC, JC, UM, LS, SD, ASL, DF, MVH, CM, BR, ES, FB, MK, JMH, GG, AP, RB, ER, LF, AP, FG, RS, CMV, AS, AL, CM, AS, MI, PP, GR, AS, LR, SR, NH, ASdT, BV, ML, SP, DG, SG, RB, MT, FM, ML, MT, AP, FZ, PQ, RG, AG, AZ, CT, AM, MB, EC, ND, AS, MB, IRC, DJP. Visualization: AC. Supervision: DJP. Project administration: AC. Funding acquisition: DJP.

**Acknowledgements:** OnCovid received direct project funding and infrastructural support by the National Institute for Health Research (NIHR) Imperial Biomedical Research Centre (BRC). D.J. Pinato is supported by grant funding from the Wellcome Trust Strategic Fund (PS3416) and acknowledges grant support from the Cancer Treatment and Research Trust (CTRT) and the Associazione Italiana per la Ricerca sul Cancro (AIRC MFAG Grant ID 25697). A. Cortellini is supported by the NIHR Imperial BRC. G. Gaidano is supported by the AIRC 5 × 1000 Grant, No. 21198, Associazione Italiana per la Ricerca sul Cancro Foundation, Milan, Italy. A. Gennari is supported by the AIRC IG Grant, No. 14230, Associazione Italiana per la Ricerca sul Cancro Foundation, Milan, Italy. A. Gennari and G. Gaidano from the University of Piemonte Orientale (Novara, Italy) acknowledge support from the UPO Aging Project.

Members of the OnCovid study group: Georgina Hanbury, Chris Chung, Meera Patel (Imperial College London, London, United Kingdom), Gino Dettorre (Washington University School of Medicine, St. Luis, United States), Christopher CT Sng, Tamara Yu, Marianne Shawe-Taylor, Hamish DC Bain, Lee Cooper, Lucy Rogers, Katherine Belessiotis, Cian Murphy, Samira Bawany, Saira Khalique, Ramis Andaleeb (University College London, London, United Kingdom), Eleanor Apthorp (King’s College London, London, United Kingdom), Roxana Reyes (Hospital Clinic Barcelona, Barcelona, Spain), David Garcia-Illescas, Nadia Saoudi (Vall dHebron University Hospital, Barcelona, Spain), Ariadna Roqué Lloveras (Catalan, Institute of Oncology, Girona, Spain), Ricard Mesia, Andrea Plaja, Marc Cucurull (Institut Català dOncologia, Badalona, Spain), Federica Grosso, Vittorio Fusco, (Ospedale Antonio e Biagio e Cesare Arrigo, Alessandria, Italy), Alice Baggi, (Azienda Ospedaliera Spedali Civili, Brescia, Italy), Maristella Saponara (Istituto Europeo di Oncologia, Milano, Italy), Luca Cantini (Universitá Politecnica delle Marche, Ancona, Italy).

**Disclaimer:** The views expressed are those of the author(s) and not necessarily those of the NIHR or the Department of Health and Social Care.

## Data Availability

The data that support the findings of this study are not publicly available. They can be made available upon reasonable to the corresponding author [AC].

## Supplementary Material

djac057_Supplementary_Data
