# Supplementary material for: COVID-19 Sequelae and the Host Proinflammatory Response: An Analysis From the OnCovid Registry
Source: J Natl Cancer Inst. 2022 Apr 13;114(7):979–87. doi: 10.1093/jnci/djac057 (PMC9047221; doi:10.1093/jnci/djac057)

Supplementary Materials

COVID-19 sequelae and the host pro-inflammatory response: an analysis from the OnCovid registry.

Cortellini A et al.

Supplementary Methods p. 2

Supplementary Table 1 p. 6
Patient disposition across participating centres.

Supplementary Table 2 p. 7
Patients and disease characteristics

Supplementary Table 3 p. 9
Inflammatory markers/indices at post COVID-19 reassessment

Supplementary Table 4 p. 10
Multivariable analyses including the interactions terms

Supplementary Figure 1 p. 11
Receiver operating characteristic (ROC) curve analysis

Supplementary Figure 2 p. 12
Post COVID-19 survival estimates according to inflammatory markers/indices

Supplementary Figure 3 p. 13
Fixed multivariable analysis for post COVID-19 risk of death

Supplementary Figure 4 p. 14
Multivariable analysis for COVID-19 sequelae among patients with advanced and non-advanced disease

Supplementary Methods

OnCovid (NCT04393974) is an active European registry study that, since the beginning of the pandemic, has collected consecutive patients fulfilling the following inclusion criteria: 1) age ≥18 years; 2) diagnosis of SARS-CoV-2 infection confirmed by RT-PCR of a nasopharyngeal swab; 3) history of solid or hematologic malignancy, at any time during the patients' past medical history, either active or in remission at the time of COVID-19 diagnosis. Patients with a history of non-invasive/premalignant lesions or with low malignant potential (i.e., basal cell carcinoma of the skin, non-invasive carcinoma in situ of the cervix, ductal carcinoma in situ) were excluded. For hematologic malignancies, only patients with a history of oncologic diseases with defined malignant behavior (lymphoma, leukaemia, multiple myeloma) were included.

OnCovid was granted central approval by the United Kingdom Health Research Authority (20/HRA/1608) and by the corresponding research ethics committees at each participating institution. Core study data were collated from electronic medical records into a case report form designed using the Research Electronic Data Capture software (REDCap, Vanderbilt University, Nashville, TN, USA). Multi-site access and data curation was coordinated by the Medical Statistics Unit in Novara, Italy.

This OnCovid registry focused on post COVID-19 outcomes among COVID-19 survivors who underwent a formal clinical reassessment at the participating institutions.

COVID-19 sequelae were defined as any residual symptoms and/or measurable organ disfunction attributable to COVID-19. The clinical definitions of symptoms, clinical syndromes, complications from COVID-19 followed criteria published by the World Health Organization (available from: https://apps.who.int/iris/bitstream/handle/10665/345824/WHO-2019-nCoV-Post-COVID-19-condition-Clinical-case-definition-2021.1-eng.pdf). These were assessed by treating physicians as per local practice and when clinically indicated, for instance during clinical consultation, symptoms review, physical examination, imaging and/or laboratory findings review.

Timing of follow-up was not standardized but dictated by the discretion of treating physicians as per standard of care.

COVID-19 sequelae were categorized according to the system/organ involved into: respiratory symptoms (including dyspnoea and chronic chough), residual fatigue, weight loss, neuro-cognitive sequelae (including cognitive, visual impairment, ano/dysosmia - age/dysgeusia, headache, confusion, lethargy), and others (including other organs disfunctions, residual fever, muscle cramps, arthralgia, skin conditions, etc).

Accounting for the unbalanced distribution of patient/disease-related features across the subgroups we adopted a fixed multivariable regression model, adjusting all estimates for clinical characteristics already known to influence clinical outcomes in patients with COVID-19 and cancer.

The following key variables of interest were used as covariates within the survival analysis:

- Sex (male vs female),
- Age (≥65 vs < 65 years),
- Number of co-morbidities (0-1 vs ≥ 2),
- Primary tumour (clustered as: breast, gastro-intestinal, gynaecological/genito-urinary, thoracic, and others),
- Tumour stage (defined as advanced vs non-advanced). In details, we defined as “advanced” stage any patient with distant metastatic disease, to differentiate them from “non-advanced” patients. Disease-specific criteria (i.e. Rai, Binet criteria etc.) were utilised as appropriate to define advanced haematological malignancies.
- Tumour status (presence of active vs non-active disease), on the basis of disease-specific criteria (radiologic, clinical, biochemical/haematological depending on disease type).
- Receipt of systemic anticancer therapy within 4 weeks of SARS-CoV-2 infection (yes vs no),

• Experience of at least one COVID-19 complications including acute respiratory failure, ARDS, kidney injury, secondary infections, sepsis, septic shock, acute cardiac injury, acute liver injury and others (yes vs no);

• Receipt of any COVID-19 specific therapy, including antivirals, antimalarials, antibiotics, corticosteroids and others (yes vs no),

• Hospitalization requirement (pre-existent/due to COVID-19 vs not required).

Being an observational study, decisions on initiating, resuming or discontinuing therapy were dictated by the discretion of the treating clinicians. The overarching subgrouping of tumour related features according to stage, activity and therapy has been consistently utilised in all the publications from our registry[1-6] ,and was made necessary by the wide heterogeneity oncological diagnoses included in the registry.

Oncological and disease specific variables were collected at baseline, defined at the moment of diagnosis of SARS-CoV-2 by PCR test. Characteristics of severity, complications and therapy against COVID-19 were collected throughout the observation period until full clinical resolution of COVID-19 or patients' mortality.

Patient observation time started from date of first PCR/SARS-CoV-2 infection confirmation until patient death or loss to follow-up. Being a retrospective, observational study, the entirety of the OnCovid cohort was followed up at intervals dictated by the routine clinical practice in each participating institutions, as deemed clinically indicated by the treating physicians. All-cause of mortality was retrieved and validated by investigators at each centre by accessing patients' electronic medical records and death certificates.

Patients were lost to follow-up when for any reason failed to attend planned follow-up appointments scheduled by the treating clinicians. Given the pragmatic nature of this registry, based on standard of care clinical practice, we could not accurately reconstruct the reasons to explain why a proportion of patients did not attend for follow-up. To avoid incurring into bias, by

mislabelling patients that were lost to follow-up as potentially deceased, we decided to exclude all patients with incomplete/missing follow up data to preserve the integrity of our results.

References

1. Dettorre GM, Dolly S, Loizidou A, et al. Systemic pro-inflammatory response identifies patients with cancer with adverse outcomes from SARS-CoV-2 infection: the OnCovid Inflammatory Score. *J Immunother Cancer* 2021; 9(3).
2. OnCovid Study G, Pinato DJ, Patel M, et al. Time-Dependent COVID-19 Mortality in Patients With Cancer: An Updated Analysis of the OnCovid Registry. *JAMA Oncol* 2021.
3. Pinato DJ, Lee AJX, Biello F, et al. Presenting Features and Early Mortality from SARS-CoV-2 Infection in Cancer Patients during the Initial Stage of the COVID-19 Pandemic in Europe. *Cancers (Basel)* 2020; 12(7).
4. Pinato DJ, Scotti L, Gennari A, et al. Determinants of enhanced vulnerability to coronavirus disease 2019 in UK patients with cancer: a European study. *Eur J Cancer* 2021; 150: 190-202.
5. Pinato DJ, Tabernero J, Bower M, et al. Prevalence and impact of COVID-19 sequelae on treatment and survival of patients with cancer who recovered from SARS-CoV-2 infection: evidence from the OnCovid retrospective, multicentre registry study. *Lancet Oncol* 2021; 22(12): 1669-80.
6. Pinato DJ, Zambelli A, Aguilar-Company J, et al. Clinical portrait of the SARS-CoV-2 epidemic in European cancer patients. *Cancer Discov* 2020.

Tables

Supplementary Table 1. Patient disposition across participating centres.

Institution	Patients
	No (%)
Imperial College London, London (UK)	7 (0.5)
University College London, London (UK)	112 (8.4)
Barts Health NHS Trust, London (UK)	57 (4.3)
Chelsea and Westminster Hospital, London (UK)	119 (8.9)
Guy’s and St Thomas’ NHS Foundation Trust, London (UK)	83 (6.2)
Velindre Cancer Centre, Cardiff (UK)	8 (0.6)
Hospital Clinic, Barcelona (Spain)	36 (2.7)
Vall d’Hebron University Hospital, Barcelona (Spain)	139 (10.4)
ICO Girona (Spain)	43 (3.2)
ICO Badalona (Spain)	44 (3.3)
ICO L’Hospitalet, L’Hospitalet de Llobregat, Barcelona (Spain)	60 (4.5)
Hospital de Manresa (Spain)	26 (1.9)
Palma de Mallorca Hospital, Palma de Mallorca, (Spain)	3 (0.2)
Hospital Universitario 12 de Octubre, Madrid (Spain)	12 (0.9)
University of Munich (Germany)	18 (1.3)
Institut Jules Bordet, Brussels (Belgium)	13 (1.0)
Institut Gustave Roussy, Villejuif (France)	124 (9.3)
Ospedale Maggiore della Carità, Novara (Italy)	66 (4.9)
Policlinico San Matteo, Pavia (Italy)	39 (2.9)
Humanitas Cancer Centre, Milan (Italy)	85 (6.4)
Ospedale Antonio e Biagio e Cesare Arrigo, Alessandria (Italy)	30 (2.2)
Università Campus Bio-Medico, Rome (Italy)	17 (1.3)
Ospedale Papa Giovanni XXIII, Bergamo (Italy)	55 (4.1)
Fondazione Poliambulanza Istituto Ospedaliero, Brescia (Italy)	8 (0.6)
Istituto Tumori, Milan (Italy)	6 (0.4)
Azienda Istituti Ospitalieri di Cremona, Cremona (Italy)	20 (1.5)
Azienda Ospedaliera Spedali Civili, Brescia (Italy)	-
Ospedali Riuniti di Ancona, Universitá Politecnica delle Marche (Italy)	11 (0.8)
University of Bari 'Aldo Moro', Bari (Italy)	3 (0.2)
Careggi University Hospital, Florence (Italy)	7 (0.5)
IRCCS AOU San Martino, Genova (Italy)	33 (2.5)
University of L’Aquila, L’Aquila (Italy)	24 (1.8)
Santa Maria Goretti Hospital, Latina (Italy)	8 (0.6)
Istituto Europeo di Oncologia, Milano (Italy)	10 (0.7)
Azienda Ospedaliera S. Andrea, Rome (Italy)	3 (0.2)
Azienda Ospedaliera S Maria, Terni (Italy)	10 (0.7)
Total	1339

Supplementary Table 2: Distribution of baseline patients, tumour and COVID-19 characteristics among the re-assessed patients according to COVID-19 sequelae experiencing

Patients' characteristics	Overall study population No. (%)	Without COVID-19 Sequelae No. (%)	With COVID-19 Sequelae No. (%)	P^a
Total No.	1339	136	203	
Country				
United Kingdom	386 (28.8)	323 (28.4)	63 (31.0)	.003
Spain	363 (27.1)	292 (25.7)	71 (35.0)	
Italy	435 (32.5)	378 (33.3)	57 (28.1)	
France/Belgium/Germany	155 (12.6)	143 (12.6)	12 (5.9)	
Sex				
Male	617 (46.1)	507 (44.7)	110 (54.5)	.011
Females	720 (53.9)	628 (55.3)	92 (45.5)	
Missing	2	1	1	
Age				
<65 years	678 (50.8)	590 (52.1)	88 (43.3)	.021
≥65 years	657 (49.2)	542 (47.9)	115 (56.7)	
Missing	4	4	-	
Comorbidities				
0-1	824 (61.5)	724 (63.7)	100 (49.3)	.001
≥2	515 (38.5)	412 (36.3)	103 (50.7)	
Smoking history				
Never smokers	608 (53.9)	534 (55.9)	74 (43.0)	1.002
Former/current smokers	520 (46.1)	422 (44.1)	98 (57.0)	
Missing	211	180	31	
Primary Tumour				
Breast	360 (27.2)	314 (28.0)	46 (22.7)	.028
Gastrointestinal	255 (19.3)	212 (18.9)	43 (21.2)	
Gynaecological/Genito-Urinary	297 (22.4)	246 (22.0)	51 (25.1)	
Thoracic	188 (14.2)	149 (13.3)	39 (19.2)	
Others	223 (16.9)	199 (17.8)	24 (11.8)	
Missing	16	16	-	
Tumour stage				
Local/loco-regional	675 (51.8)	572 (51.9)	103 (51.8)	.98
Advanced	627 (48.2)	531 (48.1)	96 (48.2)	
Missing	37	33	4	
Tumour status at COVID-19 diagnosis				
Remission/non measurable disease	444 (33.5)	379 (33.6)	65 (33.0)	.86
Active malignancy	880 (66.5)	748 (66.4)	132 (67.0)	
Missing	15	9	6	
Systemic Anticancer therapy at COVID-19 diagnosis[b]				
No	870 (67.5)	727 (66.5)	143 (73.3)	.33
Chemotherapy (± combos)	262 (20.3)	229 (20.9)	33 (16.9)	
ICI only regimens	53 (4.1)	44 (4.0)	9 (4.6)	

Endocrine therapy	32 (2.5)	28 (2.6)	4 (2.1)	
TKIs and MABs	50 (3.9)	45 (4.1)	5 (2.6)	
PARPi and CDKi	22 (1.7)	21 (1.9)	1 (0.5)	
Missing	50	42	8	
COVID-19 therapy				
No	641 (47.9)	571 (50.3)	70 (34.5)	< .001
Yes	698 (52.1)	565 (49.7)	133 (65.5)	
Complicated COVID-19				
No	1010 (75.4)	918 (80.8)	92 (45.3)	< .001
Yes	329 (24.6)	218 (19.2)	111 (54.7)	
Hospitalization				
Not required	469 (35.2)	443 (39.2)	26 (12.8)	
Required	591 (44.4)	447 (39.6)	144 (70.9)	< .001
Pre-existing	272 (20.4)	239 (21.2)	33 (16.3)	
Missing	7	7	-	

[a] 2-sided P values calculated with the Pearson χ2 test.

[b] Within 4 weeks of COVID-19 diagnosis.

Supplementary Table 3: Median values of inflammatory markers/indices at post COVID-19 reassessment, with their categorical distribution.[a]

Inflammatory markers	No. of patients	Median (IQR)	< median value No. (%)	≥ median value No. (%)
Post COVID-19 CRP, mg/L	348	5.0 (1.2-25.3)	205 (58.9)	143 (41.1)
Post COVID-19 LDH, UI/L	353	244 (188-345)	222 (62.9)	131 (37.1)
Post COVID-19 NLR	862	2.7 (1.71-5.1)	506 (58.7)	356 (41.3)
Post COVID-19 PLR	855	174 (120-269)	517 (60.5)	338 (39.5)
Post COVID-19 OIS	534	40 (35-43)	101 (18.9)	433 (81.1)

[a] COVID-19 = Coronavirus Disease 2019; NLR = Neutrophil-to-lymphocyte ratio; PLT = Platelet-to-lymphocyte ratio; CRP = C-reactive protein; LDH = Lactate Dehydrogenase; OIS = OnCovid Inflammatory Score; IQR = Inter Quartile Range.

Supplementary Table 4: Multivariable analyses including the interactions terms between SACT regimens at COVID-19 and primary tumours, tumour stage and tumour status.[a]

Post COVID-19 survival	Multivariable Analysis 1	Multivariable Analysis 2	Multivariable Analysis 3
	OR (95%CI)	OR (95%CI)	OR (95%CI)
SACT at COVID-19			
No	1	1	1
Chemotherapy (± combos)	0.44 (0.26-0.76)	0.61 (0.37-1.01)	0.59 (0.34-1.00)
ICI only regimens	0.26 (0.06-1.11)	1.13 (0.35-3.60)	0.89 (0.26-3.08)
Endocrine therapy	0.23 (0.05-0.99)	0.67 (0.19-2.36)	0.58 (0.14-2.46)
TKIs and MABs	0.04 (0.01-0.68)	0.84 (0.13-5.43)	0.52 (0.07-3.78)
PARPi and CDKi	0.07 (0.01-0.69)	0.37 (0.02-6.28)	0.20 (0.01-4.09)
Primary tumour			
Breast	1	1	1
Gastrointestinal	0.82 (0.46-1.46)	0.85 (0.48-1.53)	0.85 (0.48-1.52)
Gynaecological/Genito-Urinary	0.77 (0.43-1.39)	0.84 (0.47-1.51)	0.84 (0.47-1.50)
Thoracic	0.75 (0.39-1.43)	0.88 (0.47-1.65)	0.89 (0.48-1.67)
Other	0.50 (0.25-1.01)	0.61 (0.31-1.18)	0.61 (0.31-1.18)
SACT at COVID-19 * Primary tumour	P^b = .06	-	-
Tumour stage			
Local/loco-regional	1	1	1
Advanced	0.97 (0.62-1.51)	1.02 (0.64-1.62)	0.95 (0.61-1.48)
SACT at COVID-19 * Tumour stage	-	P^b = .41	-
Tumour status			
Remission/non measurable disease	1	1	1
Active malignancy	1.55 (0.97-2.48)	1.54 (0.96-2.45)	1.57 (0.97-2.54)
SACT at COVID-19 * Tumour status	-	-	P^b = .82

[a]The three columns summarise separate multivariable analyses including the same adjusting covariates but with the interaction terms included separately. Adjusting covariates for each analysis were: sex (male vs female), age (≥ 65 vs < 65 years), number of co-morbidities (0-1 vs ≥ 2), the experience of at least one COVID-19 complication (yes vs no), the receipt of any COVID-19 specific therapy (yes vs no), hospitalization (pre-existing for whatever cause, including cancer vs due to COVID-19 vs not required) and country (United Kingdom, Spain, Italy and France/Belgium/Germany). COVID-19 = Coronavirus Disease 2019; SACT: systemic anticancer therapy; ICIs = immune checkpoint inhibitors; TKIs = tyrosine kinase inhibitors; MABs = monoclonal antibodies; PARPi = poly adenosine diphosphate-ribose polymerase inhibitors; CDKi = cyclin dependent kinase inhibitors; OR=odds ratio; CI = confidence intervals.
[b] 2-sided P values calculated with Cox regressions.

Supplementary Figure 1. Receiver operating characteristic (ROC) curve analysis for COVID-19 sequelae according to baseline inflammatory markers/indices. **A)** CRP (878 patients included): AUC = 0.66 (95%CI: 0.63-0.69), p < .001; optimal cut-off: 36.7 mg/L. **B)** LDH (540 patients included): AUC = 0.57 (95%CI: 0.52-0.61), p = .028); optimal cut-off: 463 UI/L. **C)** NLR (1017 patients included): AUC = 0.58 (95%CI: 0.55 – 0.61), p < .001; optimal cut-off: 5.7. **D)** PLR (995 patients included): AUC 0.51 (95%CI: 0.47-0.54), p = .70; optimal cut-off: 455. **E)** OIS (690 patients included): AUC = 0.52 (95%CI: 0.48 – 0.56), p = .34; optimal cut-off: 42. **F)** ROC curves comparison (300 patients included). CRP: AUC 0.66 (95%CI: 0.61-0.71), LDH: AUC 0.61 (95%CI: 0.55 – 0.66), NLR: AUC 0.59 (95%CI: 0.53-0.64), OIS: AUC 0.52 (95%CI: 0.46-0.58), PLR: AUC 0.52 (95%CI: 0.46-0.57).
Difference between areas: CRP vs LDH = 0.05, SE: 0.05 (95%CI: -0.06-0.16), p = .57, CRP vs NLR = 0.07, SE: 0.05 (95%CI: -0.03-0.17), p = .18, CRP vs OIS = 0.14, SE: 0.05 (95%CI: 0.03-0.25), p = .012, CRP vs PLR = 0.14, SE: 0.05 (95%CI: 0.04-0.24), p = .007, LDH vs NLR = 0.02, SE: 0.04 (95%CI: -0.07-0.11), p = 0.69, LDH vs OIS = 0.09, SE: 0.05 (95%CI: -0.001-0.19), p = .073, LDH vs PLR = 0.09, SE: 0.05 (95%CI: -0.01-0.19), p = .08, NLR vs OIS = 0.07, SE: 0.05 (95%CI: -0.03-0.17), p = .19, NLR vs PLR = 0.07, SE: 0.03 (95%CI: 0.01-0.14), p = .040, OIS vs PLR = 0.01, SE: 0.06 (95%CI: -0.11-0.12), p = .98). COVID-19 = Coronavirus Disease 2019; NLR = Neutrophil-to-lymphocyte ratio; PLT = Platelet-to-lymphocyte ratio; CRP = C-reactive protein; LDH = Lactate Dehydrogenase; OIS = OnCovid Inflammatory Score; AUC = Area Under Curve; SE = standard error. 2-sided P-values computed with the method of De Long, et al. (1998).

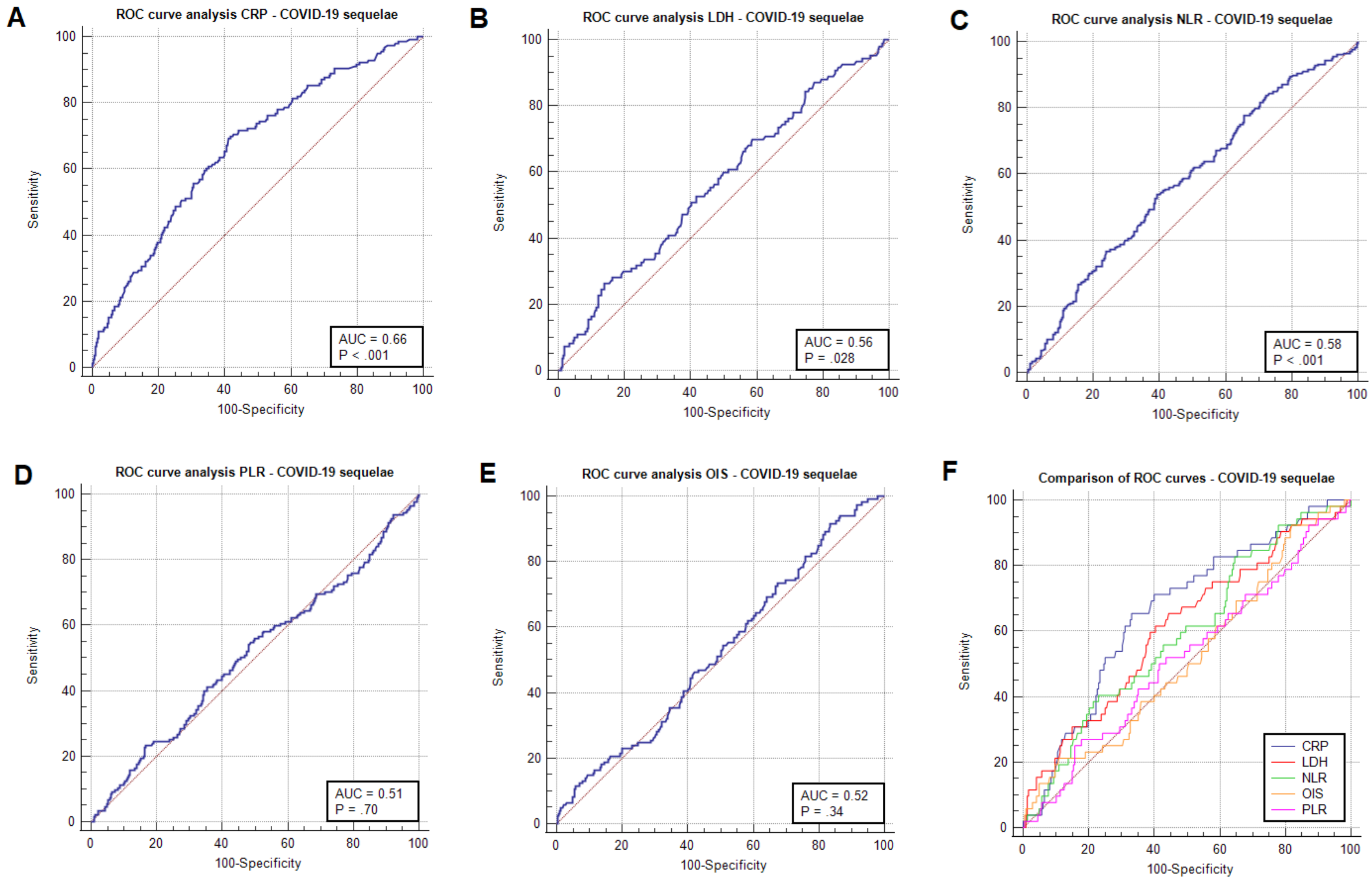

Supplementary Figure 2: post COVID-19 survival estimates according to inflammatory markers/indices. **A)** Patients with CRP < 5 mg/L: not reached (9 events) vs patients with CRP ≥ 5 mg/L: not reached (43 events). **B)** Patients with LDH < 244 UI/L: not reached (7 events) vs patients with LDH ≥ 244 UI/L: not reached (26 events). **C)** Patients with NLR < 2.7: not reached (21 events) vs patients with NLR ≥ 2.7: not reached (77 events). **D)** Patients with PLR < 174: not reached (36 events) vs patients with PLR ≥ 174: not reached (62 events). **E)** Patients with OIS ≥ 40: not reached (18 events) vs patients with OIS < 40: not reached (51 events). COVID-19 = Coronavirus Disease 2019; NLR = Neutrophil-to-lymphocyte ratio; PLT = Platelet-to-lymphocyte ratio; CRP = C-reactive protein; LDH = Lactate Dehydrogenase; OIS = OnCovid Inflammatory Score. The Kaplan-Meier method was used to estimate median survival intervals. 2-sided P values calculated with the log-rank test.

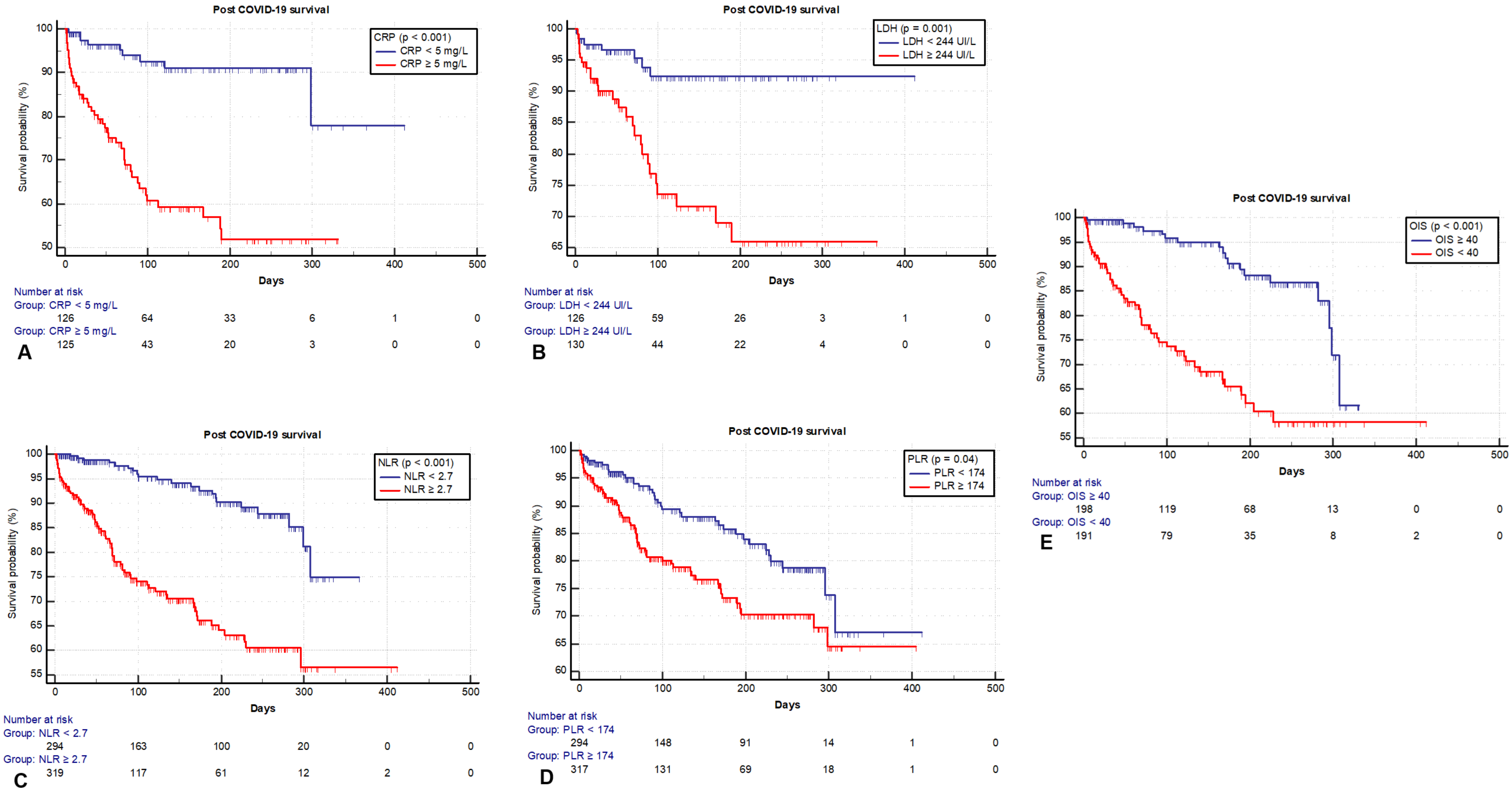

Supplementary Figure 3: fixed multivariable analysis according to categorised post COVID-19 inflammatory markers/indices for post COVID-19 risk of death. Adjusting covariates for each analysis were: gender (male vs female), age (≥ 65 vs < 65 years), number of co-morbidities (0-1 vs ≥ 2), primary tumour (clustered as: breast, gastro-intestinal, gynaecological/genito-urinary, thoracic, and others), receipt of systemic anticancer therapy (SACT) within 4 weeks of COVID-19 diagnosis (yes vs no), tumour stage (defined as advanced vs non-advanced), tumour status (presence of active vs non-active disease), experience of at least one COVID-19 complications (yes vs no), receipt of any COVID-19 specific therapy (yes vs no), hospitalization (pre-existing for whatever cause, including cancer vs due to COVID-19 vs not required) and country (United Kingdom, Spain, Italy and France/Belgium/Germany). Arrow used for CI out of the figure's range.COVID-19 = Coronavirus Disease 2019; NLR = Neutrophil-to-lymphocyte ratio; PLT = Platelet-to-lymphocyte ratio; CRP = C-reactive protein; LDH = Lactate Dehydrogenase; OIS = OnCovid Inflammatory Score; HR = hazard ratio; CI = Confidence Intervals.

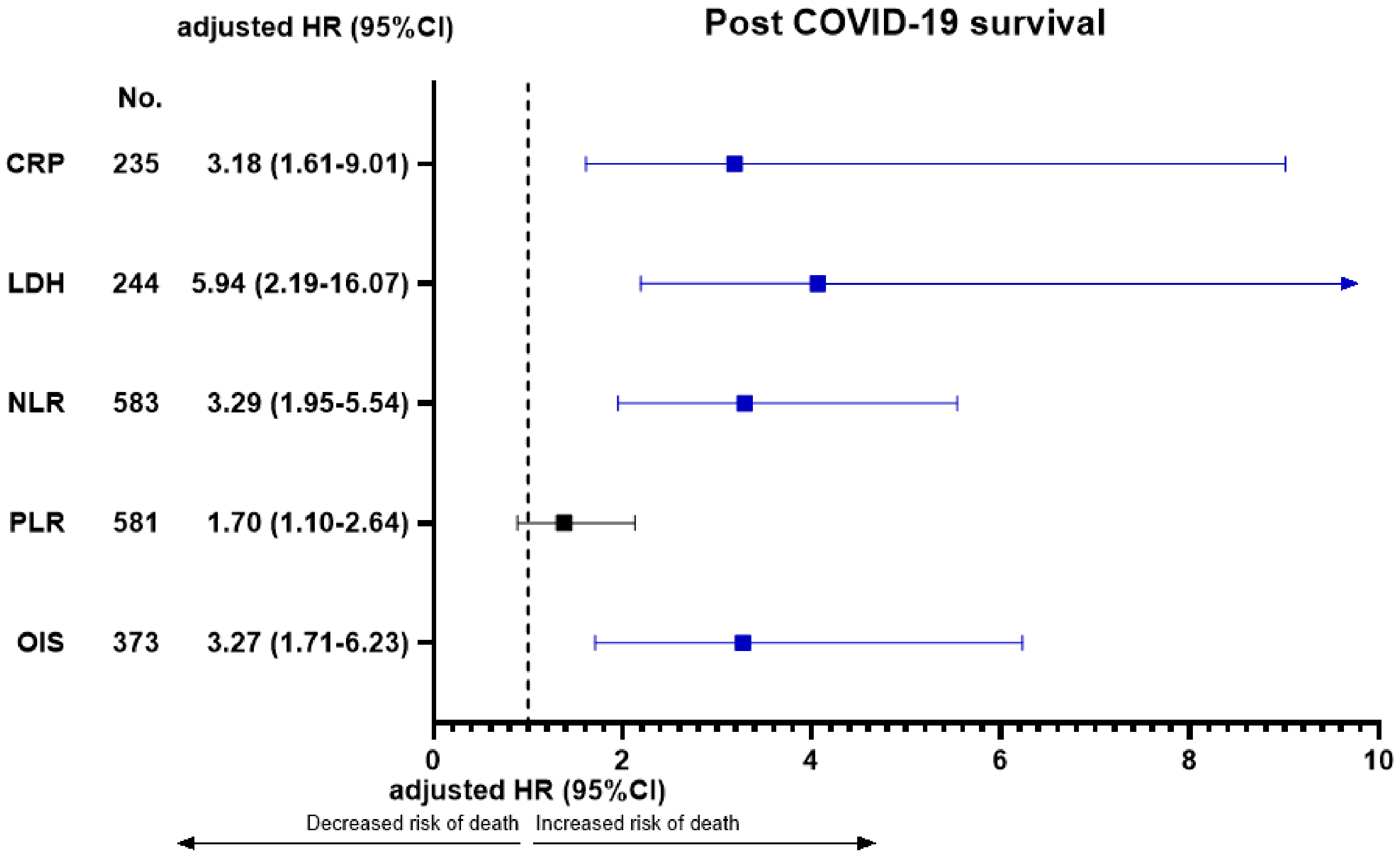

Supplementary Figure 4: fixed multivariable analysis for COVID-19 sequelae overall among patients with local/loco-regional disease **A)** and advanced disease **B)**. Arrow used for CI out of the figure's range. COVID-19 = Coronavirus Disease 2019; SACT: systemic anticancer therapy; ICIs = immune checkpoint inhibitors; TKIs = tyrosine kinase inhibitors; MABs = monoclonal antibodies; PARPi = poly adenosine diphosphate-ribose polymerase inhibitors; CDKi = cyclin dependent kinase inhibitors; GI = gastro-intestinal; GU = genito-urinary; GY = gynaecological; UK = United KingdomOR = odds ratio; CI: confidence intervals.

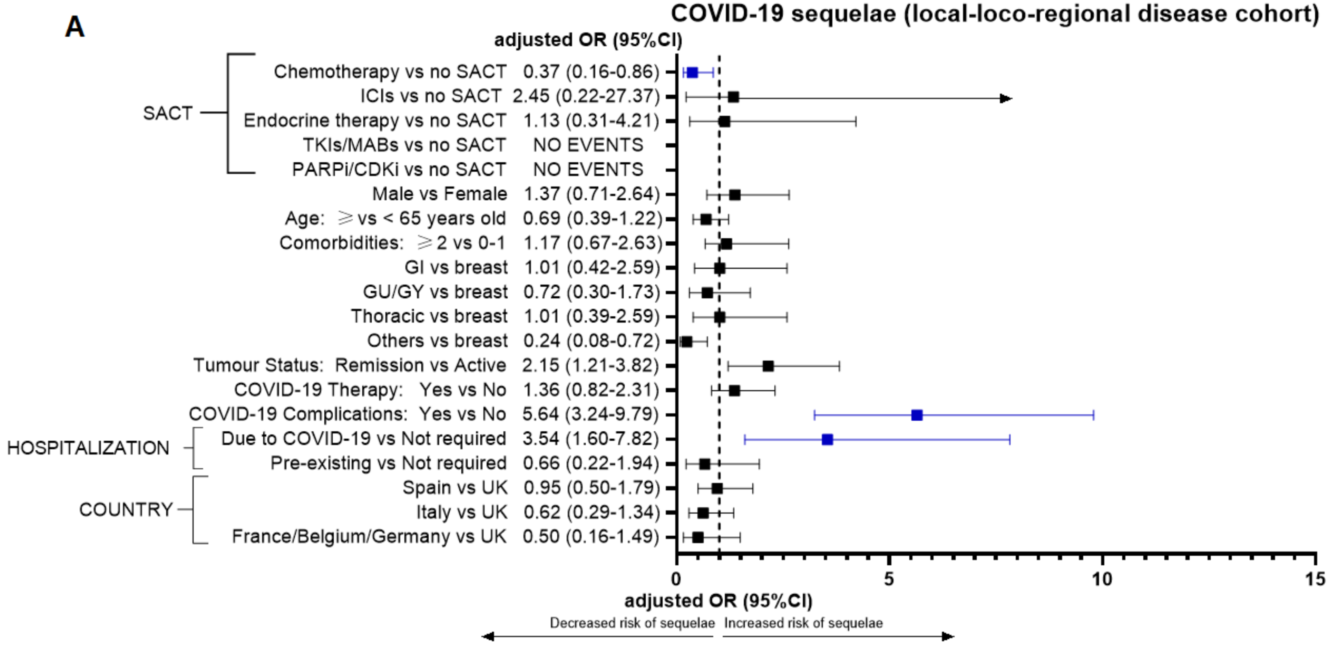

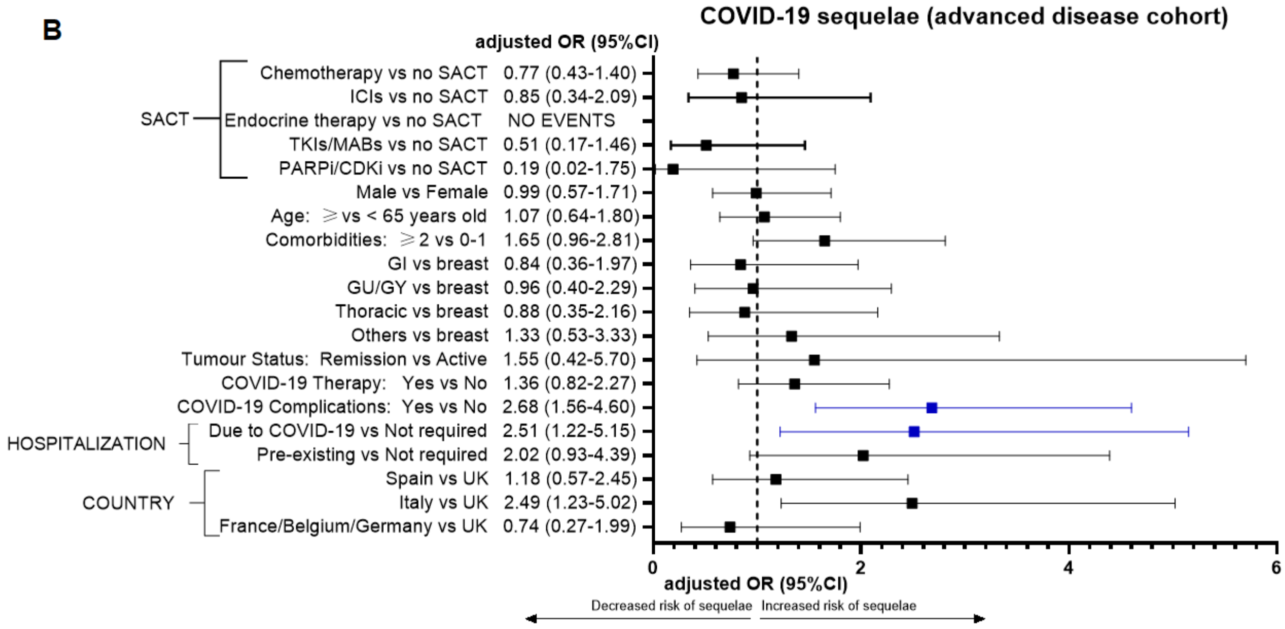

Supplement: djac057_Supplementary_Data [file djac057_supplementary_data.pdf]
